# Wounded Boys, Violent Men? Exploring Risk Factors for Intimate Partner Violence among Perpetrators with a History of Trauma

**DOI:** 10.1007/s40653-026-00942-y

**Published:** 2026-07-09

**Authors:** Cristina Expósito-Álvarez, Rocío Pérez-Sabater, Manuel Roldán-Pardo, Marisol Lila

**Affiliations:** 1 Department of Social Psychology, Faculty of Psychology and Speech Therapy, University of Valencia, Valencia, Spain; 2 Department of Basic Psychology, Neuropsychology, and Social Psychology, Faculty of Psychology, Catholic University of Valencia, Valencia, Spain

**Keywords:** Perpetrator programs, History of trauma, Intimate partner violence, Childhood trauma, Specific needs

## Abstract

One of the key challenges for enhancing the effectiveness of intervention programs for intimate partner violence (IPV) perpetrators is tailoring interventions to participants’ specific risks and needs. A history of childhood or adolescent exposure to family violence as either a direct victim or witness has been consistently associated with an increased risk of IPV perpetration in adulthood. This study examined differences between 1,126 court-mandated male IPV perpetrators with and without a history of family violence exposure during childhood or adolescence across sociodemographic, individual, social-relational, attitudinal, and violence-related variables. The study also examined whether trauma history was associated with IPV recidivism risk beyond sociodemographic characteristics and after accounting for individual, social-relational, and attitudinal factors. Compared to perpetrators without trauma exposure, those with a trauma history were younger, had lower educational attainment, included a higher proportion of immigrant participants, and reported higher impulsivity, anger, depressive symptoms, alexithymia, and substance use. They also reported more stressful life events, lower perceived family and friend support, and higher hostile sexism and victim-blaming attitudes. In addition, they reported greater physical and psychological IPV, a more advanced stage of change, and higher risk of IPV recidivism at intake. Hierarchical logistic regression analyses showed that younger age, unemployment, higher impulsivity, and lower perceived family social support were the variables most strongly associated with trauma history. Hierarchical linear regression analyses further revealed that trauma history remained significantly associated with IPV recidivism risk after accounting for covariates. These findings underscore the importance of incorporating trauma-informed strategies into IPV perpetrator interventions.

Intimate partner violence (IPV) is a public health problem of pandemic proportions (World Health Organization [WHO], 2021) that affects victims’ physical and mental health (White et al., 2024). According to a recent WHO report (2025), 25.8% of women aged 15–49 have been victims of physical and/or sexual violence by their partners or ex-partners. Several authors have researched the consequences of IPV and observed that this type of violence affects not only victims (Muñoz-Rivas et al., 2024), but also society as a whole (Walker-Descartes et al., 2021).

One of the responses to prevent this type of violence is the implementation of intervention programs for IPV perpetrators (Lila et al., 2019). Recent systematic reviews and meta-analyses suggest that the effectiveness of these programs may be enhanced when interventions are aligned with the Risk–Needs–Responsivity (RNR) model (Andrews & Bonta, 2010) and the Principles of Effective Intervention (Richards et al., 2022), which emphasize tailoring treatment to perpetrators’ specific criminogenic risks and individual treatment needs (Butters et al., 2021; Cheng et al., 2021). Within this framework, identifying the characteristics and risk profiles associated with different subgroups of IPV perpetrators is essential for improving risk assessment and developing more individualized interventions.

Among these factors, trauma experienced in childhood or adolescence is consistently recognized as one of the most robust predictors of IPV perpetration and may therefore require attention in such programs (Shorey et al., 2021; Taft et al., 2011; Zhu et al., 2024). These early adversities, commonly referred to as adverse childhood experiences (ACEs), encompass a broad range of maltreatment and family dysfunction dynamics, including physical, emotional and sexual abuse, neglect, and exposure to parental substance abuse, mental illness, or domestic violence in childhood or adolescence (Ports et al., 2020). More specifically, evidence from systematic reviews and meta-analyses indicates that being exposed to family violence during childhood or adolescence, either through direct victimization or by witnessing IPV between caregivers or other intimate partners, is associated with a greater likelihood of IPV perpetration later in life (Godbout et al., 2019; Wadji et al., 2025). The enduring effects of trauma, such as disruption of the emotional regulation system, impaired social information processing, heightened threat sensitivity, and tendency to disorganized, avoidant or insecure attachment styles, can shape later relationship functioning and are key mechanisms through which trauma may contribute to IPV perpetration (Miles-McLean et al., 2019; Taft et al., 2015, 2016).

Several theoretical models are proposed to explain the association between exposure to trauma in childhood or adolescence and IPV perpetration. The general aggression model (Anderson & Bushman, 2002) suggests that early exposure to violence contributes to the internalization of aggressive scripts and the normalization of violence as a conflict resolution strategy. This model aligns with the intergenerational transmission of violence hypothesis, which posits that witnessing or experiencing violence in childhood normalizes its use and increases the risk of replicating such behaviors in adulthood (Voith et al., 2020). The attachment theory (Bowlby, 1982) also provides insight into how early abuse and neglect can lead to maladaptive internal working models of relationships by increasing vulnerability to IPV perpetration (Green & Browne, 2019; Purnell, 2010). In addition, feminist theories highlight how trauma can intersect with patriarchal norms, which suggests that a history of trauma may amplify the drive for control and dominance in intimate relationships as a compensatory mechanism for earlier experiences of powerlessness (Gilbar et al., 2020; Lee et al., 2013). Together, these frameworks underscore the multifaceted role of exposure to IPV in childhood or adolescence in increasing the likelihood of subsequent IPV perpetration, and they highlight the importance of trauma-informed approaches in intervention programs for IPV perpetrators (Gilbar et al., 2020). A clearer understanding of the characteristics associated with trauma history among IPV perpetrators is essential for informing trauma-informed interventions and identifying participants’ specific intervention-related needs (Zhu et al., 2024).

Building on Bronfenbrenner’s (1979) ecological model, Heise (2011) conceptualized IPV as a multifactorial phenomenon associated with factors operating across individual, relational, community, and societal levels. Accordingly, IPV perpetrators who witnessed or experienced family violence during childhood or adolescence may present specific vulnerabilities across these levels. This perspective provides a useful framework for organizing the multiple domains examined in the present study and for understanding how vulnerabilities associated with trauma history may be reflected across different areas relevant to IPV intervention. For example, sociodemographic characteristics may reflect broader contextual conditions and accumulated disadvantages that are relevant to participants’ life circumstances and relationship dynamics and may help characterize differences between IPV perpetrators with and without a history of childhood or adolescent trauma exposure (Meyer et al., 2024).

At the individual level, characteristics such as poor emotional regulation, particularly difficulties in managing anger and impulsivity, are key features of trauma responses, often characterized by hyperarousal, mistrust and heightened emotional reactivity (Li et al., 2020). These difficulties can impair an individual’s ability to control violent impulses by increasing the likelihood of hostility and aggression during interpersonal conflict (Green & Browne, 2019). For instance, difficulties in identifying and expressing feelings, commonly referred to as alexithymia, have been linked to a higher risk of IPV, especially when co-occurring with other risk factors like depressive symptoms or substance use among IPV perpetrators (Lawrence et al., 2023; Maloney et al., 2023). Although prior research highlights these associations, less is known about how such emotional and psychological difficulties may differ among IPV perpetrators with and without trauma histories (Maguire et al., 2015).

In addition to psychological adjustment difficulties, substance use, including alcohol, cocaine and cannabis, represents another key individual-level domain frequently associated with IPV perpetration (Cafferky et al., 2018; Langenderfer, 2013). The literature reveals that IPV perpetrators with substance use problems have a higher incidence of exposure to family violence in childhood or adolescence than IPV perpetrators without substance use problems (Alexander, 2014; Expósito-Álvarez et al., 2021, 2023; Siria et al., 2021a, 2021b). Therefore, it remains crucial to examine the role of substance use among trauma-exposed individuals participating in intervention programs for IPV perpetrators (Rivas-Rivero & Bonilla-Algovia, 2022; Travers et al., 2022). This could help to inform the development of targeted, trauma-informed strategies that address this group of participants’ specific needs (McKenna & Holtfreter, 2021).

Beyond individual-level vulnerabilities, social-relational variables also warrant attention, as trauma exposure may be associated with difficulties in interpersonal functioning among IPV perpetrators with a history of childhood or adolescent trauma exposure. For example, stressful life events, particularly those related to family or relationship conflict, could trigger or escalate violent behaviors more strongly in those individuals who are witnesses of family violence early in life (Roberts et al., 2011a, 2011b). Conversely, perceived social support can have a buffering effect on trauma symptoms by potentially lowering the likelihood of IPV among individuals with a trauma history (Hoskins & Kunkel, 2022). Examining these variables could provide insights into the social contexts that either exacerbate or protect against IPV perpetration in perpetrators with a trauma history.

In addition to interpersonal functioning, trauma history may also be reflected in attitudes and beliefs relevant to IPV perpetration. Attitudes toward women, including sexism and victim-blaming, may also be critical in understanding IPV perpetration, especially in individuals with a trauma history, who may have normalized controlling and abusive behaviors toward women (Gilbar et al., 2020). Therefore, examining these variables may help clarify differences between IPV perpetrators with and without childhood or adolescent trauma exposure.

Finally, IPV perpetrators with a trauma history, specifically those who were victims of or witnessed family violence in childhood or adolescence, represent a high-risk group of participants within intervention programs for IPV perpetrators (St-Pierre Bouchard et al., 2023; Voith et al., 2020). In addition to the domains discussed above, violence-related variables, such as self-reported IPV, stage of change, and facilitator-assessed risk of IPV recidivism, may provide valuable information regarding participants’ acknowledgment of their violent behavior and inform more targeted intervention strategies (Green & Browne, 2019).

To further explore these relationships, the present study examined a broad range of variables across multiple social-ecological domains among IPV perpetrators with and without a history of direct victimization or exposure to family violence during childhood or adolescence. These variables included sociodemographic characteristics, individual factors, such as psychological adjustment and substance use, social-relational and attitudinal variables, and violence-related characteristics assessed at baseline. Specifically, the study had two primary objectives: (1) to identify the characteristics that distinguish IPV perpetrators with and without a history of childhood or adolescent trauma exposure, and to determine which factors most strongly distinguish these groups within a hierarchical multivariable framework; and (2) to examine whether trauma history is associated with increased risk of IPV recidivism at intake beyond sociodemographic characteristics, and whether this association remains significant after accounting for individual, social-relational, and attitudinal variables. Together, these objectives may contribute to identifying key intervention targets relevant to improving outcomes among this high-risk subgroup of IPV perpetrators.

## Method

### Participants

The study sample comprised 1,126 male court-mandated participants who attended a community-based intervention program for IPV perpetrators at the University of Valencia (*Contexto* *Program*; Lila et al., 2018). The study enrolled the participants in one of the 104 intervention groups, with each comprising 9–12 participants. Groups were convened between June 2006 and June 2024. The inclusion criteria were as follows: (1) being at least 18 years old, (2) having been convicted of IPV against a female partner or ex-partner under Spanish Organic Law 1/2004 on Integrated Protection Measures against Gender Violence (Organic Law 1/2004, 2004) and being court-mandated to attend an intervention program for IPV perpetrators as a condition of sentence suspension, and (3) signing the informed consent form. Individuals were excluded if they presented severe mental health problems, severe cognitive impairment, or disruptive behavior that interfered with the assessment or intervention sessions. Table 1 presents the sample’s sociodemographic characteristics.

**Table 1:** Sample characteristics

Variables	Total sample: *n* = 1,126		
	M(SD)	Range	*n*(%)
Age	40.5 (11.7)	18–81	
Annual income^1^	4.39 (2.36)	1–12	
Origin			
Spain			812 (71.9)
Latin America			157 (13.9)
Europe (except Spain)			88 (7.9)
Africa			57 (5.2)
Asia			10 (0.9)
Middle East			2 (0.2)
Immigrant status			
No			812 (71.9)
Yes			314 (28.1)
Level of education			
No education			89 (7.90)
Elementary			539 (47.9)
High School			398 (35.3)
College			100 (8.9)
Relationship status			
Without a partner			848 (75.3)
With a partner			278 (24.7)
Employment			
Yes			664 (59.0)
No			462 (41.0)

*M:* Mean, *SD:* Standard Deviation.

^1^Annual income: 1: <€1,800, 2: €1,800-€3,600, 3: €3,600-€6,000, 4: €6,000-€12,000, 5: €12,000-€18,000, 6: €18,000-€24,000, 7: €24,000-€30,000, 8: €30,000-€36,000, 9: €36,000-€60,000, 10: €60,000–€90,000, 11: €90,000–€120,000 and 12: >€120,000.

### The Intervention Program

Participants were enrolled in the *Contexto* *Program*, a community-based, court-mandated intervention for men convicted of IPV and sentenced to less than two years in prison, whose sentence had been suspended conditional on program participation (Lila et al., 2018). The program is delivered in a closed-group format and implemented at the University of Valencia, Spain.

The intervention spans approximately 11 months and comprises a total of 70 h, including 35 weekly two-hour group sessions and five individual sessions, each lasting approximately one hour. Groups consist of 9 to 12 participants and are facilitated by two trained psychologists. As a closed program, no new participants are admitted to a group once the intervention has started.

The program is grounded in an integrative theoretical framework that combines the ecological model (Heise, 2011), cognitive-behavioral model, and a feminist perspective, in line with WHO (2012). Its primary aim is to reduce risk factors and enhance protective factors associated with IPV perpetration through evidence-based cognitive-behavioral and motivational strategies.

The group intervention is structured into five modules addressing key domains related to IPV. For example, the program includes components aimed at improving emotional regulation and anger management, enhancing relational skills, and addressing broader social and attitudinal factors, including gender roles, sexist beliefs, and social support. Throughout the group intervention, participants are also asked to complete homework activities designed to reinforce and apply the content addressed during the sessions, which are reviewed in the following session. In addition, the program incorporates an Individualized Motivational Plan (IMP; Lila et al., 2018), which is designed to enhance treatment engagement and motivation to change. The IMP is based on several evidence-based approaches aimed at fostering motivation and engagement, including motivational interviewing (Miller & Rollnick, 2012), the stages of change model (Prochaska et al., 1992), solution-focused brief therapy (Bannink, 2007; De Shazer & Berg, 1997), the Good Lives Model (Langlands et al., 2009), and the therapeutic alliance framework (Bordin, 1979). It includes five individual motivational interviews (three at intake to establish personal goals, one mid-program to review progress, and one at the end to assess goal attainment), which also provide an opportunity for facilitators to gather relevant information about participants (e.g., the presence or absence of a history of trauma in childhood or adolescence). In addition, the IMP includes three group sessions in which participants share progress and achievements toward their goals and receive feedback and support from facilitators and other group members. Facilitators continuously reinforce participants’ goals throughout the intervention.

### Instruments

#### Sociodemographic Characteristics

Data were collected on age, immigrant status (0 = *no*; 1 = *yes*), relationship status (0 = without a partner; 1 = with a partner), level of education (1 = *no education*; 2 = *elementary studies*; 3 = *high school studies*; 4 = *college studies*), employment (0 = *no*; 1 = *yes*), and annual income (from 1 = *less than €1,800* to 12 = *more than €120,000*).

#### Individual Variables: Psychological Adjustment

##### Anger

We employed the State-Trait Anger Expression Inventory (STAXI-2; Spielberger, 1999; Spanish version by Miguel-Tobal et al., 2001). STAXI-2 is a 49-item inventory with responses given on a 4-point Likert-type scale (1 = *not at all* to 4 = *very much*). The inventory assesses state anger as a situational response, and trait anger as an inherent disposition. An overall Anger Expression Index (AEI) is also provided. The Spanish version demonstrates good test-retest reliability (Miguel-Tobal et al., 2001) and is used with IPV perpetrators (Osa-Subtil et al., 2024; Siria et al. 2021a, 2021b). Cronbach’s α coefficients for state anger, trait anger and AEI were 0.89, 0.82 and 0.69, respectively.

##### Depressive Symptomatology

We used the Center for Epidemiologic Studies Depression Scale-7 (CESD-7; Radloff, 1977; Spanish and short version by Herrero & Gracia, 2007). The CESD-7 evaluates the frequency and intensity of depressive symptomatology over the past week. The scale comprises seven items, all rated on a 4-point Likert-type scale (1 = *rarely* to 4 = *all the time or most of the time*). The Spanish short version has been previously employed in IPV perpetrators samples (Expósito-Álvarez et al., 2025; Lila et al., 2019). In the present study, Cronbach’s α coefficient was 0.88.

##### Impulsivity

We used the Plutchik Impulsivity Scale (PIS; Plutchik & Van Praag, 1989; Spanish version by Páez et al., 1996). The PIS assesses participants’ impulsivity level through 15 items on a 4-point Likert-type scale (1 = *never* to 4 = *almost always*). The Spanish version has been utilized in IPV perpetrators samples (Lila et al., 2019; Osa-Subtil et al., 2024; Romero-Martínez et al., 2013). Cronbach’s α coefficient was 0.71.

##### Alexithymia

We applied the Toronto Alexithymia Scale-20 Items (TAS-20; Bagby et al., 1994; Spanish version by Martínez-Sánchez, 1996). The TAS-20 is a 20-item instrument administered on a 6-point Likert-type scale (1 = *strongly disagree* to 6 = *strongly agree*). This study utilizes only two subscales: difficulty identifying feelings and difficulty describing feelings. The TAS-20 has demonstrated adequate reliability and validity (Bagby et al., 1994). The Spanish version has been utilized in IPV perpetrators samples (Expósito-Álvarez et al., 2025; Romero-Martínez et al., 2019). In this study, Cronbach’s coefficients were 0.88 and 0.74 for the subscales of difficulty identifying feelings and difficulty describing feelings, respectively.

#### Individual Variables: Substance Use

##### Alcohol Use

We administered the Alcohol Use Disorders Identification Test (AUDIT; Babor & Grant, 1989; Spanish version by Contel et al., 1999). The AUDIT is a well-validated screening measure that evaluates the frequency and quantity of alcohol use. The scale consists of 10 items, each rated on a 4-point Likert-type scale (for example, 1 = *never* to 4 = *daily or almost daily*). The AUDIT has been extensively utilized in research studies involving IPV populations in Spain (Catalá-Miñana et al., 2013; Romero-Martínez et al., 2023). Cronbach’s coefficient was 0.81 in the present study.

##### Cannabis and Cocaine Use

We used the Severity of Dependence Scale (SDS; Miele et al., 2000; Spanish version by Vélez-Moreno et al., 2013). The SDS independently assesses cocaine and cannabis dependence over the past 12 months. Each subscale comprises five items on a 4-point Likert-type scale (0 = *never* to 3 = *always*). The SDS has demonstrated criterion validity (Gossop et al., 1995). Its Spanish version has been previously employed with IPV male perpetrators (Expósito-Álvarez et al. 2024a; Sarrate-Costa et al., 2022). In this study, the Cronbach’s coefficients were 0.87 for cannabis use and 0.83 for cocaine use.

#### Social-relational Variables

##### Stressful Life Events

We employed the Stressful Life Events Questionnaire (SLEQ; Gracia & Herrero, 2004). Participants were asked to identify the stressful events they had experienced in the past six months from a list of 33 events (0 = *not present*, 1 = *present*). The SLEQ has demonstrated satisfactory reliability coefficients and has been utilized with Spanish IPV perpetrators (Expósito-Álvarez et al., 2024b; Lila et al. 2014). The Cronbach’s α coefficient was 0.72.

##### Perceived Social Support

We applied the Multidimensional Scale of Perceived Social Support (MSPSS; Zimet et al., 1990; Spanish version by Calderón et al., 2021). The MSPSS is a 12-item scale with responses given on a 7-point Likert-type scale (1 = *very strongly disagree* to 7 = *very strongly agree*). The scale includes three subscales: family members, friends and significant others. The MSPSS has demonstrated good internal reliability (Zimet et al., 1990). Cronbach’s α coefficients for family members, friends and significant others were 0.92, 0.82 and 0.91, respectively.

#### Attitudes Toward IPV

##### Sexism

We used the Ambivalent Sexism Inventory (ASI; Glick & Fiske, 1997; Spanish version by Expósito et al., 1998). The ASI encompasses 22 items, with responses measured on a 6-point Likert-type scale (0 = *strongly disagree* to 5 = *strongly agree*). The inventory features two subscales: benevolent and hostile sexism. The ASI has been utilized in studies involving Spanish IPV perpetrators (Expósito-Álvarez et al. 2024b; Juarros-Basterretxea et al. 2018). The Cronbach’s α coefficients for benevolent and hostile sexism were 0.85 and 0.90, respectively.

##### Victim-blaming

We employed the Victim-Blaming Attitudes in cases of Intimate Partner Violence Against Women (VB-IPVAW; Martín-Fernández et al., 2018). The VB-IPVAW comprises 12 items on a 4-point Likert-type scale (1 = *strongly disagree* to 4 = *strongly agree*). This scale has demonstrated strong reliability and validity, and it has been utilized in IPV perpetrators samples (Martín-Fernández et al., 2022). Cronbach’s α coefficient was 0.89.

#### Violence-related Variables

##### Self-reported IPV

We utilized the Revised Conflict Tactics Scale (CTS-2; Straus et al., 1996; Spanish version by Loinaz et al., 2012) to evaluate occurrence of violence against partners within the past 12 months. The present study solely employs the physical and psychological subscales, with responses measured on an 8-point Likert-type scale (0 = *this has never happened* to 6 = *more than 20 times in the past year*, and 7 = *never in the past year*,* but it has happened before*) using the frequency-based scoring method proposed by Straus et al. (1996). To address the impact of asymmetric and skewed distributions, extreme outliers are subjected to truncation, a method first proposed by Smamash (1981). This approach has been used in similar studies (Expósito-Álvarez et al., 2024a; Lee et al., 2022; Marshall et al., 2021). The CTS-2 has proven evidence for construct and discriminant validity (Straus et al., 1996). Its Spanish version has been widely used with IPV perpetrators samples (Lila et al., 2018; Loinaz et al., 2012; Osa-Subtil et al., 2024). Cronbach’s coefficients for the physical and psychological subscales were 0.70 and 0.76, respectively.

##### Stage of Change

In accordance with the Transtheoretical Model of Change (Prochaska & DiClemente, 1982), facilitators evaluated participants’ stage of change. Stage of change coding is as follows: 1 = *precontemplation*, 2 = *contemplation*, 3 = *preparation*, 4 = *action* and 5 = *maintenance*. This procedure has been employed in previous studies involving Spanish IPV perpetrators samples (Lila et al., 2025; Roldán-Pardo et al., 2025).

##### Risk of IPV Recidivism Assessed by the Facilitators

We used the Spousal Assault Risk Assessment Guide (SARA; Kropp et al., 1999; Spanish version by Andrés-Pueyo et al., 2008). The SARA is a widely used 20-item clinical checklist on a 3-point Likert-type scale (0 = *absent*, 1 = *possibly present*, and 2 = *present*) that includes the main IPV risk factors. The SARA was completed by facilitators at intake based on available case information, including court records and individual interviews. An overall score was used as the indicator of total IPV recidivism risk at intake, with higher total scores representing greater risk of IPV recidivism. The SARA possessed predictive validity (Messing & Thaller, 2013), and its Spanish version has been extensively used with IPV perpetrators (Expósito-Álvarez et al., 2025; Lila et al., 2018; Llor-Esteban et al., 2016).

### Procedure

We conducted the present study in accordance with the 1964 Declaration of Helsinki. Participants provided written informed consent in the assessment and motivation phase and were informed that their decision to participate in this study and their decision to decline would have no impact on their legal status or grant them any legal benefits. We collected all the data as a part of the initial assessment for the participants enrolled in the *Contexto* *Program* (Lila et al., 2018). We obtained these data using a self-reported assessment battery, administered by the program staff for two 2-hour assessment sessions. We assessed the data on the risk of IPV by the facilitators after the assessment and motivation phase prior to group commencement. We collected all the data in accordance with the procedures approved by the Committee of Ethics and Human Research of the University of Valencia (H1537520365110). After collecting all data, participants were classified into two groups: those with a history of trauma and those without. Trauma history was coded as present when participants explicitly verbalized exposure to family violence in childhood or adolescence. Participants who scored above 0 on the SARA were classified as having a history of trauma (*n* = 330), whereas those who scored 0 were classified as having no history of trauma (*n* = 796). See Expósito-Álvarez et al. (2025) for a similar procedure.

### Data Analysis

We first conducted univariate analyses to compare participants with and without a trauma history across the study variables, following a social-ecological approach: (1) sociodemographic variables, (2) individual variables, including psychological adjustment and substance use variables, (3) social-relational variables, (4) attitudes toward IPV, and (5) violence-related variables. For dichotomous variables, chi-square tests were conducted and effect sizes were estimated using Cramér’s *V*. Values above 0.10, 0.30, and 0.50 were interpreted as small, moderate, and large effect sizes, respectively (Cohen, 1988; Rea & Parker, 1992). For continuous variables, one-way ANOVAs with Welch’s correction were performed because this procedure is more robust when the assumption of homogeneity of variances is violated and group sizes are unequal (Delacre et al., 2017; Fagerland & Sandvik, 2009; Ruxton, 2006). Effect sizes were estimated using eta squared, with values above 0.01, 0.06, and 0.14 interpreted as small, moderate, and large effect sizes, respectively (Levine & Hullett, 2002). Because multiple univariate tests were conducted, *p*-values were adjusted within each set of variables using the false discovery rate (FDR) procedure to reduce the likelihood of type I error. The FDR procedure is designed to control the expected proportion of significant results by penalizing the *p*-values associated with the null hypothesis (Benjamini, 2010; Benjamini & Hochberg, 1995).

To further address the first study objective, and after identifying group differences through univariate analyses, a hierarchical binary logistic regression analysis was conducted to determine which variables most strongly distinguished IPV perpetrators with and without a history of childhood or adolescent trauma exposure within a multivariable framework. Trauma history was entered as the dependent variable. Variables were entered into four sequential blocks: (1) sociodemographic characteristics, (2) individual variables, (3) social-relational and attitudinal variables, and (4) violence-related variables. To support the inclusion of relevant covariates, variables were considered eligible for the multivariable models if they were associated with trauma history in the univariate analyses at *p* <.10 or were considered clinically relevant, following recommendations for purposeful variable selection in regression modeling (Bursac et al., 2008; Hosmer et al., 2013). When conceptually overlapping variables were identified, only one variable was retained based on theoretical relevance and strength of association with the outcome to reduce redundancy and potential multicollinearity. Model fit was evaluated using likelihood ratio chi-square tests, −2 log likelihood values, and Nagelkerke’s *R²*. Adjusted odds ratios (*ORs)*, 95% CIs, and Wald statistics were reported for each variable included in the model.

To address the second study objective, a hierarchical linear regression analysis was conducted to examine whether trauma history was associated with IPV recidivism risk at intake. The total SARA score was entered as the dependent variable, with higher scores indicating greater risk of IPV recidivism. Variables were entered in four sequential blocks: (1) sociodemographic characteristics, (2) trauma history, (3) individual variables, and (4) social-relational and attitudinal variables. This approach allowed us to examine whether trauma history was associated with IPV recidivism risk beyond sociodemographic characteristics, and whether this association remained significant after accounting for individual, social-relational, and attitudinal variables. Model fit and incremental explanatory value were evaluated using *R²*, adjusted *R²*, changes in *R²*, and *F*-tests. Unstandardized coefficients, standard errors, standardized coefficients, *t* statistics, and *p*-values were reported for all variables included in the model. Regression assumptions and multicollinearity diagnostics were examined for both models. All statistical analyses were conducted using IBM SPSS Statistics, version 28.0.1.1.

### Trauma History

Trauma history was assessed using Item 6 of the *Spousal Assault Risk Assessment Guide* (SARA; Kropp et al., 1999; Spanish version by Andrés-Pueyo et al., 2008), which evaluates whether participants were direct victims of and/or witnessed family violence during childhood or adolescence (0 = *no*, 1 = *yes*). Facilitators assessed this information during the individual motivational interviews.

## Results

### What Characteristics Distinguish Participants with and without a History of Trauma?

We first examined the differences between the participants with and without trauma in different sets of variables, following a social-ecological approach. Table 2 presents the descriptive statistics for the two groups.

**Table 2:** Comparative of participants with and without trauma at multiple levels

Variables	W Trauma (*n* = 330)		W/O Trauma (*n* = 796)		*F/χ* ^*2*^	*p*	*η²/V*
	*M/n*	*SD/%*	*M/n*	*SD/%*			
Sociodemographic characteristics							
Age	38.5	11.4	41.3	11.7	12.9	< .001	0.011
Immigrant status					4.12	.042	0.061
No	221	67.6	585	73.6			
Yes	106	32.4	210	26.4			
Relationship status					0.05	.817	0.007
Without a partner	247	74.8	601	75.5			
With a partner	83	25.2	195	24.5			
Level of education					10.2	.017	0.095
No education	28	8.5	61	7.7			
Elementary studies	179	53.3	363	45.6			
High school studies	108	32.7	290	36.4			
College studies	18	5.5	82	10.3			
Employment					3.28	.070	0.054
No	149	45.2	313	39.3			
Yes	181	54.8	483	60.7			
Annual income	4.26	2.16	4.44	2.44	1.49	.233	0.001
Individual variables: Psychological adjustment							
State anger	17.3	4.75	16.7	3.50	4.89	.033	0.006
Trait anger	17.5	5.80	15.6	4.92	25.3	< .001	0.026
AEI	25.5	12.3	22.7	11.0	13.3	< .001	0.013
Depression	2.17	0.86	1.87	0.78	30.6	< .001	0.029
Impulsivity	2.02	0.42	1.83	0.38	45.2	< .001	0.043
TAS-20 I	2.74	1.22	2.29	1.21	16.1	< .001	0.029
TAS-20 E	3.15	1.21	2.73	1.17	14.3	< .001	0.027
Individual variables: Substance use							
Alcohol use	6.68	7.04	4.78	5.37	18.9	< .001	0.021
Cannabis use	1.96	3.38	0.85	2.21	15.2	< .001	0.037
Cocaine use	1.37	2.88	0.65	1.98	8.84	.005	0.021
Social-relational variables							
Stressful life events	3.56	3.25	2.83	2.79	12.3	< .001	0.013
MSPSS family	5.25	1.74	5.80	1.60	18.5	< .001	0.023
MSPSS friends	4.96	1.67	5.52	1.77	19.4	< .001	0.021
MSPSS significant others	5.45	1.70	5.69	1.61	3.97	.051	0.005
Attitudes toward IPV							
Benevolent sexism	2.69	1.18	2.60	1.14	1.27	.261	0.001
Hostile sexism	2.49	1.21	2.29	1.20	5.72	.023	0.006
Victim blaming	0.67	0.56	0.56	0.51	4.95	.033	0.009
Violence-related variables							
CTS physical	3.66	8.08	1.63	4.95	14.7	< .001	0.029
CTS psychological	9.13	11.1	5.49	9.08	22.3	< .001	0.023
Stage of change	1.24	0.49	1.16	0.42	4.58	.037	0.006
Risk of IPV	12.9	5.58	8.37	4.64	167.9	< .001	0.148

*W:* with, *W/O:* without, *M:* mean, *SD:* standard deviation, *F:* F-value, χ^2^: chi-square test, *p:* *p*-value, *η²*: eta square, *V:* Cramér’s *V*, *AEI:* Anger Expression Index, *TAS-20 I:* Toronto Alexithymia Scale-20 difficulty identifying feelings, *TAS-20 E:* Toronto Alexithymia Scale-20 difficulty expressing feelings, *MSPSS:* Multidimensional Scale of Perceived Social Support, *IPV:* intimate partner violence, *CTS:* Conflict Tactics Scale

With respect to sociodemographic characteristics, participants with trauma were younger (*F* = 12.9, *p* <.001, *η²* = 0.011) and had a lower level of education (*χ*^*2*^ = 10.2, *p* =.017, *V* = 0.095). Specifically, they showed a higher rate of elementary studies and a lower rate of college studies compared to those without trauma. Additionally, a higher proportion of immigrant participants showed a history of trauma (*χ*^*2*^ = 4.12, *p* =.042, *V* = 0.061). Although these differences were statistically significant, effect sizes were generally small, with age showing a small effect and immigrant status and education level showing negligible effects (*V* < 0.10). No significant differences appeared in relationship status, employment and annual income.

In terms of the individual variables, participants with trauma reported higher levels of state and trait anger (*F* = 4.75, *p* =.033, *η²* = 0.006 and *F* = 25.3, *p* <.001, *η²* = 0.026, respectively), anger expression index (*F* = 13.3, *p* <.001, *η²* = 0.013), impulsivity (*F* = 45.2, *p* <.001, *η²* = 0.043), depressive symptomatology (*F* = 30.6, *p* <.001, *η²* = 0.029), and difficulties in identifying and expressing feelings (*F* = 16.1, *p* <.001, *η²* = 0.029; *F* = 14.3, *p* <.001, *η²* = 0.027; respectively). The effect size for state anger was negligible (*η²* < 0.01), whereas the remaining variables showed a small effect size. However, the effect size for impulsivity approached a moderate level (*η²* = 0.043). Similarly, in terms of substance use, participants with trauma reported higher levels of alcohol (*F* = 18.9, *p* <.001, *η²* = 0.021), cannabis (*F* = 15.2, *p* <.001, *η²* = 0.037) and cocaine use (*F* = 8.84, *p* =.005, *η²* = 0.021) compared to those without trauma history. All the substance use variables had small effect sizes.

Regarding social-relational variables, participants with trauma reported a higher incidence of stressful life events (*F* = 12.3, *p* <.001, *η²* = 0.013) and lower perceived social support from family members and friends (*F* = 18.5, *p* <.001, *η²* = 0.023; *F* = 19.4, *p* <.001, *η²* = 0.021, respectively) compared to those without trauma. All the variables had small effect sizes. No significant differences were observed in the perceived social support provided by significant others.

In relation to attitudes toward IPV, the participants with trauma reported higher levels of victim-blaming attitudes (*F* = 4.95, *p* =.033, *η²* = 0.009) and hostile sexism (*F* = 5.72, *p* =.023, *η²* = 0.006) compared to those without a history of trauma. Both effect sizes were negligible (*η²* < 0.01). No significant differences emerged for benevolent sexism.

With respect to violence-related variables, the participants with trauma reported greater perpetration of physical and psychological violence (*F* = 14.7, *p* <.001, *η²* = 0.029; *F* = 22.3, *p* <.001, *η²* = 0.023, respectively), were on a more advanced stage of change (*F* = 4.58, *p* =.037, *η²* = 0.006), and a higher risk of IPV recidivism (*F* = 167.9, *p* <.001, *η²* = 0.148) than those without a history of trauma. Effect sizes were small, except for the risk of IPV, which was large.

Secondly, a hierarchical binary logistic regression analysis was conducted to determine which variables most strongly distinguished participants with a history of trauma from those without trauma exposure. Variables were entered into four sequential blocks: (1) sociodemographic characteristics, (2) individual variables, (3) social-relational and attitudinal variables, and (4) violence-related variables. The results for each model are presented in Table 3.

**Table 3:** Results of the hierarchical logistic regression analysis on trauma history (*n* = 468)

Variable	Model 1			Model 2			Model 3			Model 4		
	B(SE)	Wald	OR[95%CI]	B(SE)	Wald	OR[95%CI]	B(SE)	Wald	OR[95%CI]	B (SE)	Wald	OR[95%CI]
Age	− 0.04(0.01)***	14.6	0.97[0.95, 0.98]	− 0.02(0.01)*	5.67	0.98[0.96, 0.99]	− 0.03(0.01)**	7.16	0.97[0.95, 0.99]	− 0.02(0.01)*	4.97	0.98[0.96, 0.99]
Immigrant status	0.33(0.24)	1.82	1.39[0.86, 2.23]	0.39(0.26)	2.24	1.48[0.89, 2.46]	0.37(0.27)	1.91	1.45[0.86, 2.47]	0.38(0.27)	1.96	1.46[0.86, 2.49]
Relationship status	− 0.29(0.25)	1.47	0.74[0.46, 1.20]	− 0.28(0.26)	1.21	0.75[0.45, 1.25]	− 0.29(0.27)	1.15	0.75[0.45, 1.27]	− 0.25(0.27)	0.85	0.78[0.46, 1.32]
Level of education	− 0.29(0.14)*	4.38	0.74[0.56, 0.98]	− 0.21(0.15)	1.94	0.81[0.61, 1.09]	− 0.18(0.16)	1.37	0.83[0.61, 1.13]	− 0.17(0.16)	1.21	0.84[0.62, 1.15]
Employment	− 0.71(0.22)***	10.5	0.49[0.32, 0.75]	− 0.63(0.23)**	7.34	0.53[0.34, 0.84]	− 0.59(0.23)*	6.22	0.56[0.35, 0.88]	− 0.57(0.24)*	5.77	0.56[0.35, 0.90]
Trait anger				0.01(0.02)	0.26	1.01[0.97, 1.06]	0.01(0.02)	0.13	1.01[0.96, 1.05]	0.01(0.02)	0.08	1.01[0.96, 1.06]
Depression				0.15(0.14)	1.03	1.16[0.87, 1.54]	0.06(0.15)	0.15	1.06[0.79, 1.43]	0.07(0.15)	0.22	1.08[0.79, 1.45]
Impulsivity				1.01(0.32)**	9.92	2.74[1.46, 5.13]	0.91(0.33)**	7.71	2.48[1.31, 4.72]	0.84(0.33)*	6.44	2.33[1.21, 4.46]
Difficulty identifying feelings				0.02(0.11)	0.03	1.02[0.83, 1.25]	0.01(0.11)	0.01	1.01[0.81, 1.24]	− 0.01(0.11)	0.01	0.99[0.80, 1.23]
Alcohol use				0.01(0.02)	0.35	1.01[0.98, 1.05]	0.01(0.02)	0.38	1.01[0.98, 1.05]	0.01(0.02)	0.45	1.01[0.98, 1.05]
Cannabis use				0.05(0.04)	1.14	1.05[0.96, 1.14]	0.05(0.04)	1.31	1.05[0.97, 1.15]	0.05(0.04)	1.19	1.05[0.96, 1.15]
Cocaine use				0.02(0.05)	0.13	1.02[0.92, 1.12]	0.01(0.05)	0.06	1.01[0.92, 1.12]	0.01(0.05)	0.06	1.01[0.92, 1.12]
Stressful life events							0.02(0.04)	0.20	1.02[0.94, 1.11]	0.01(0.04)	0.03	1.01[0.93, 1.09]
MSPSS family							− 0.17(0.06)**	7.94	0.84[0.74, 0.95]	− 0.18(0.06)**	8.15	0.84[0.74, 0.95]
Hostile sexism							− 0.06(0.11)	0.34	0.94[0.76, 0.95]	− 0.09(0.11)	0.64	0.92[0.74, 1.14]
Victim blaming							0.23(0.24)	0.88	1.25[0.78, 2.01]	0.20(0.26)	0.68	1.22[0.76, 1.98]
Stage of change										0.01(0.27)	0.01	1.00[0.59, 1.71]
CTS psychological										0.02(0.01)	2.37	1.02[0.99, 1.05]
CTS physical										0.01(0.02)	0.01	1.00[0.96, 1.04]
*R*^*2*^ Nagelkerke			0.09			0.17			0.19			0.20
*ΔR*^*2*^ Nagelkerke						0.08			0.02			0.01
χ²			29.5***			59.5***			69.1***			72.2***
−2 Log Likelihood			566.3			536.2			526.7			523.6

*SE:* standard error, *OR:* odds ratio, *CI:* confidence interval, *MSPSS:* Multidimensional Scale of Perceived Social Support, *CTS:* Conflict Tactics Scale.

*ΔR*^*2*^ Nagelkerke Model 2 = *R*^*2*^ Nagelkerke Model 2 - *R*^*2*^ Nagelkerke Model 1; *ΔR*^*2*^ Nagelkerke Model 3 = *R*^*2*^ Nagelkerke Model 3 - *R*^*2*^ Nagelkerke Model 2; *ΔR*^*2*^ Nagelkerke Model 4 = *R*^*2*^ Nagelkerke Model 4 - *R*^*2*^ Nagelkerke Model 3.

*p <.05, **p <.01, ***p <.001

In Model 1, sociodemographic characteristics explained 9% of the variance in trauma history (Nagelkerke’s *R²* = 0.09). Participants with a trauma history tended to be younger (*B* = − 0.04, *SE* = 0.01, *p* <.001, *OR* = 0.97), have a lower educational level (*B* = − 0.29, *SE* = 0.14, *p* =.036, *OR* = 0.74), and be less likely to be employed (*B* = − 0.71, *SE* = 0.22, *p* <.001, *OR* = 0.49) than participants without trauma exposure. In Model 2, the inclusion of individual variables increased the proportion of explained variance to 17% (Nagelkerke’s *R²* = 0.17, Δ*R²* = 0.08). Impulsivity emerged as one of the strongest distinguishing variables between participants with and without a trauma history (*B* = 1.01, *SE* = 0.32, *p* =.002, *OR* = 2.74). Employment (*B* = − 0.63, *SE* = 0.23, *p* =.007, *OR* = 0.53) and age (*B* = − 0.02, *SE* = 0.01, *p* =.017, *OR* = 0.98) remained significant. In Model 3, social-relational and attitudinal variables were added, resulting in a modest increase in explained variance (Nagelkerke’s *R²* = 0.19, Δ*R²* = 0.02). Impulsivity (*B* = 0.91, *SE* = 0.33, *p* =.005, *OR* = 2.48), employment (*B* = − 0.59, *SE* = 0.23, *p* =.013, *OR* = 0.56), and age (*B* = − 0.03, *SE* = 0.01, *p* =.007, *OR* = 0.97) remained significant. In addition, perceived family social support was associated with lower odds of trauma history (*B* = − 0.17, *SE* = 0.06, *p* =.005, *OR* = 0.84).

Finally, Model 4 yielded a small increase in explained variance (Nagelkerke’s *R²* = 0.20, Δ*R²* = 0.01). The overall pattern of findings remained stable. Impulsivity (*B* = 0.84, *SE* = 0.33, *p* =.011, *OR* = 2.33), perceived family social support (*B* = − 0.18, *SE* = 0.06, *p* =.004, *OR* = 0.84), employment (*B* = − 0.57, *SE* = 0.24, *p* =.016, *OR* = 0.56), and age (*B* = − 0.02, *SE* = 0.01, *p* =.026, *OR* = 0.98) remained significantly associated with trauma history. Overall, compared with participants without a trauma history, those with a trauma history tended to be younger, report higher impulsivity, have lower perceived family social support, and be less likely to be employed.

### Is Trauma History Associated with Increased Risk of IPV Recidivism?

A hierarchical linear regression analysis was conducted to examine variables associated with risk of IPV recidivism at intake. Variables were entered into four sequential blocks: (1) sociodemographic characteristics, (2) trauma history, (3) individual variables, and (4) social-relational and attitudinal variables. The results for each model are presented in Table 4.

**Table 4:** Results of the hierarchical linear regression analysis on risk of IPV recidivism (*n* = 474)

Variable	Model 1			Model 2			Model 3			Model 4		
	B(SE)	β	t	B(SE)	β	t	B(SE)	β	t	B(SE)	β	t
Age	− 0.10(0.02)	− 0.24	−5.31***	− 0.08(0.02)	− 0.18	−4.20***	− 0.05(0.02)	− 0.13	−2.88***	− 0.06(0.02)	− 0.13	−3.16***
Immigrant status	0.13(0.52)	0.01	0.26	− 0.10(0.49)	− 0.01	− 0.21	− 0.04(0.47)	− 0.01	− 0.08	− 0.20(0.48)	− 0.02	− 0.42
Relationship status	− 0.44(0.51)	− 0.04	− 0.85	− 0.28(0.48)	− 0.02	− 0.59	− 0.15(0.46)	− 0.01	− 0.32	− 0.08(0.46)	− 0.01	− 0.17
Level of education	−1.21(0.29)	− 0.19	−4.17***	− 0.99(0.27)	− 0.15	−3.65***	− 0.76(0.26)	− 0.12	−2.86***	− 0.59(0.27)	− 0.09	−2.17*
Employment	−2.24(0.47)	− 0.22	−4.77***	−1.69(0.45)	− 0.17	−3.79***	−1.44(0.43)	− 0.14	−3.36***	−1.40(0.43)	− 0.14	−3.28***
Trauma history				3.52(0.44)	0.34	8.03***	2.65(0.43)	0.25	6.13***	2.58(0.43)	0.25	5.95***
Trait anger							0.02(0.04)	0.02	0.38	− 0.01(0.05)	− 0.01	− 0.28
Depression							0.49(0.27)	0.08	1.85	0.31(0.28)	0.05	1.10
Impulsivity							1.10(0.58)	0.09	1.89	1.07(0.59)	0.09	1.81
Difficulty identifying feelings							0.20(0.19)	0.05	0.99	0.10(0.20)	0.02	0.48
Alcohol use							0.05(0.03)	0.06	1.39	0.04(0.03)	0.05	1.11
Cannabis use							0.27(0.08)	0.15	3.31***	0.26(0.08)	0.14	3.12***
Cocaine use							0.12(0.09)	0.06	1.29	0.13(0.09)	0.06	1.38
Stressful life events										0.11(0.08)	0.06	1.43
MSPSS family										− 0.10(0.12)	− 0.04	− 0.88
Hostile sexism										0.47(0.20)	0.11	2.38*
Victim blaming										− 0.05(0.44)	− 0.01	− 0.11
*R* ^*2*^			0.12			0.23			0.32			0.34
*ΔR* ^*2*^						0.11			0.09			0.02
Adjusted *R*^*2*^			0.11			0.22			0.30			0.31
*F*			13.1***			64.5***			8.76***			2.52*

*SE:* standard error, *MSPSS:* Multidimensional Scale of Perceived Social Support.

Δ*R*^*2*^ Model 2 = *R*^*2*^ Model 2 - *R*^*2*^ Model 1; *ΔR*^*2*^ Model 3 = *R*^*2*^ Model 3 - *R*^*2*^ Model 2; *ΔR*^*2*^ Model 4 = *R*^*2*^ Model 4 - *R*^*2*^ Model 3.

*p <.05, **p <.01, ***p <.001

In Model 1, sociodemographic variables explained 11% of the variance in IPV recidivism risk (adjusted *R²* = 0.11). Age (*B* = − 0.10, *SE* = 0.02, *p* <.001), level of education (*B* = −1.21, *SE* = 0.29, *p* <.001), and employment status (*B* = −2.24, *SE* = 0.47, *p* <.001) were negatively associated with IPV recidivism risk. In Model 2, the inclusion of trauma history significantly improved the model, increasing the explained variance to 22% (adjusted *R²* = 0.22; Δ*R²* = 0.11). Trauma history was positively associated with IPV recidivism risk (*B* = 3.52, *SE* = 0.44, *p* <.001). Age, level of education, and employment remained significantly associated with IPV recidivism risk, although their effects were slightly attenuated. In Model 3, individual variables were added, further increasing the explained variance to 30% (adjusted *R²* = 0.30; Δ*R²* = 0.09). Trauma history remained significantly associated with IPV recidivism risk (*B* = 2.65, *SE* = 0.43, *p* <.001). Age, level of education, and employment also remained significant. Among the individual variables, cannabis use was the only variable significantly and positively associated with IPV recidivism risk (*B* = 0.27, *SE* = 0.08, *p* <.001).

Finally, in Model 4, social-relational and attitudinal variables were included, yielding a modest but statistically significant increase in explained variance (adjusted *R²* = 0.31; Δ*R²* = 0.02). Trauma history (*B* = 2.58, *SE* = 0.43, *p* <.001) and cannabis use (*B* = 0.26, *SE* = 0.08, *p* =.002) remained significantly and positively associated with IPV recidivism risk. Age (*B* = − 0.06, *SE* = 0.02, *p* =.002), level of education (*B* = − 0.59, *SE* = 0.27, *p* =.030), and employment (*B* = −1.40, *SE* = 0.43, *p* =.001) continued to show significant negative associations with IPV recidivism risk. Additionally, hostile sexism was positively associated with IPV recidivism risk (*B* = 0.47, *SE* = 0.20, *p* =.018).

Overall, the hierarchical regression analysis showed that trauma history was significantly associated with higher IPV recidivism risk beyond sociodemographic characteristics and remained significantly associated with IPV recidivism risk after accounting for individual, social-relational, and attitudinal variables. In addition, higher cannabis use, stronger hostile sexist attitudes, younger age, lower educational attainment, and unemployment were also associated with higher IPV recidivism risk.

## Discussion

The present study examined a broad range of sociodemographic, individual, social-relational, attitudinal, and violence-related characteristics among IPV perpetrators with and without a history of childhood or adolescent trauma exposure. First, the study aimed to identify the characteristics that distinguished these groups and to determine which factors most strongly distinguished them within a hierarchical multivariable framework. Second, we examined whether trauma history was associated with risk of IPV recidivism at intake beyond sociodemographic characteristics and after accounting for individual, social-relational, and attitudinal variables. To the best of our knowledge, this is the first study to adopt such a comprehensive social-ecological approach among IPV perpetrators who experienced or witnessed family violence during childhood or adolescence to inform intervention strategies sensitive to the specific needs of trauma-exposed IPV perpetrators.

Regarding the first study objective, the univariate analyses identified several differences between IPV perpetrators with and without a history of childhood or adolescent trauma exposure across the social-ecological domains examined. With respect to the sociodemographic characteristics, our results revealed that participants with a history of trauma, unlike those without, were younger, had a lower level of education and included a higher proportion of immigrant participants. However, when these variables were considered within the hierarchical multivariable framework, only younger age and unemployment remained significantly associated with trauma history, although employment had not reached statistical significance in the univariate analyses. This suggests that some sociodemographic differences observed at the univariate level may overlap with other individual, social-relational and attitudinal variables, or violence-related characteristics, and that the relevance of employment status may become more apparent when considered alongside these factors. The association between trauma history and younger age may reflect the relevance of developmental timing and early adversity in shaping the risk profiles of IPV perpetrators (Jiang et al., 2025). In addition, the association with unemployment suggests that trauma-exposed perpetrators may experience greater current social or economic vulnerability, reflecting broader psychosocial needs during intervention (Yeo et al., 2024).

At an individual level, the participants with trauma reported higher levels of impulsivity, state and trait anger, and had a higher expression index, compared to those without trauma. This is consistent with the literature showing that perpetrators with more anger problems are also, and to a greater extent, witnesses to or victims of violence in their childhood compared to the participants with fewer anger problems (Eckhardt et al., 2008). These impairments in managing impulsivity and anger may increase the propensity for violent behavior, especially among individuals who are already at elevated risk due to their exposure to violence in childhood or adolescence (Maguire et al., 2015; Semiatin et al., 2017). Importantly, impulsivity emerged as one of the variables most strongly associated with trauma history in the hierarchical regression models, even after accounting for sociodemographic, social-relational, attitudinal, and violence-related variables. Our results also revealed that IPV perpetrators with a trauma history showed higher levels of depressive symptomatology than those without. This is consistent with the study by Siria et al. (2021a, 2021b), which revealed that those participants who experienced childhood family violence scored higher for major depression. Our results also showed that participants with a trauma history had lower emotion regulation skills, particularly difficulties in identifying and expressing feelings, which is consistent with previous studies (Expósito-Álvarez et al., 2025; Swopes et al., 2013). It is possible to explain these results by the fact that early experiences of trauma contribute to dysregulated nervous systems, which can impair emotion regulation processes and facilitate violent behavior later in life (Spencer et al., 2024). Moreover, and alignment with previous studies (Expósito-Álvarez et al., 2021, 2023; Gilchrist et al., 2019), the participants with a history of trauma reported higher levels of alcohol, cannabis and cocaine use. Emerging evidence suggests that individuals with a history of trauma, particularly in childhood or adolescence, may more likely engage in substance use as a maladaptive coping mechanism to manage the emotional dysregulation and psychological distress associated with unresolved trauma (Lawrence et al., 2023).

At a social-relational level, the IPV perpetrators with a history of trauma reported a higher incidence of stressful life events compared to those without. This pattern aligns with research which suggests that early exposure to trauma, defined as any stressful experience that overwhelms one’s sense of safety and control, can increase vulnerability to later adversity and a greater likelihood of IPV perpetration (Voith et al., 2020). Our results also revealed that participants with trauma perceived lower levels of social support from their family and friends compared to those without, but no significant differences emerged for support from significant others. Given that trauma may originate within the family system, individuals exposed to childhood family violence may exhibit more mistrust of close family relatives and friends. This mistrust may reduce their help-seeking behavior, as they may feel they should process their emotions independently, thereby limiting the buffering effects of social support (Hoskins & Kunkel, 2022). Moreover, lower perceived family social support remained significantly associated with trauma history in the hierarchical regression analysis, underscoring the relevance of family relational dynamics among trauma-exposed perpetrators.

Our univariate results showed that, compared to perpetrators without trauma, those with a trauma history exhibited significantly higher levels of victim-blaming attitudes and hostile sexism, whereas no differences emerged for benevolent sexism. These findings are consistent with the intergenerational transmission of violence framework (Voith et al., 2020), which suggests that early exposure to violence may be associated with maladaptive cognitive and relational patterns. In this regard, witnessing or experiencing IPV in childhood may be linked to distorted cognitive schemas, including a greater tendency to attribute responsibility for violent behavior to the victim (Gilbar et al., 2020). Similarly, difficulties in emotion identification and regulation, particularly anger management, may be associated with more hostile sexist attitudes (Norlander & Eckhardt, 2005). However, because these attitudinal variables did not remain significant in the multivariable model, these findings should be interpreted as group differences rather than as independent factors distinguishing perpetrators with and without trauma histories.

Regarding violence-related variables, participants with a trauma history reported greater perpetration of both physical and psychological IPV, a more advanced stage of change, and higher IPV recidivism risk than those without trauma exposure. However, self-reported IPV perpetration and stage of change did not remain significantly associated with trauma history in the final hierarchical regression model. This suggests that differences in violence-related characteristics may partly overlap with other factors that more clearly distinguished trauma-exposed perpetrators, particularly higher impulsivity, lower perceived family social support, younger age, and unemployment. Therefore, although the univariate findings are consistent with the notion that early exposure to violence may be linked to later violent behavior in intimate relationships (Roberts et al. 2011a, 2011b; Voith et al. 2020), the multivariable results point to a broader risk profile in which individual and relational vulnerabilities may play a more central role.

Regarding the second study objective, the findings showed that trauma history was associated with IPV recidivism risk after accounting for sociodemographic characteristics, and that this association remained significant after the subsequent inclusion of individual, social-relational, and attitudinal variables. In addition, higher cannabis use, stronger hostile sexist attitudes, younger age, lower educational attainment, and unemployment were also associated with greater IPV recidivism risk. Taken together, these findings suggest that trauma-exposed perpetrators may present a more complex risk profile, characterized by psychosocial, behavioral, and attitudinal needs that should be considered when designing targeted, evidence-based intervention strategies (Travers et al., 2021; Zhu et al., 2024).

Therefore, our results have important treatment implications because they underscore the importance of developing trauma-informed interventions adjusted to the specific risks and needs identified at multiple levels among IPV perpetrators. Although trauma-informed approaches have been more commonly implemented among veteran populations (Creech et al., 2018; Taft et al., 2016), trauma-informed IPV interventions have shown promise in reducing IPV recidivism risk among IPV perpetrators (Karakurt et al., 2019; McKenna & Holtfreter, 2021; Travers et al., 2021). In this context, trauma-informed approaches do not necessarily imply trauma-focused treatment, but rather the development of intervention environments that are sensitive to participants’ trauma histories, promote emotional safety and awareness, strengthen personal and relational resources, and avoid retraumatization while maintaining accountability for violent behavior (Scott & Jenney, 2023). Given the challenges involved in therapeutically exploring trauma histories within group-based intervention programs for IPV perpetrators (Wilson et al., 2015), the present findings may help inform areas in which existing programs could become more responsive to trauma-exposed participants. These include being sensitive and responsive to participants’ socio-economic background, addressing impulsivity and substance use, enhancing the quality of their social support networks, and helping participants recognize and reconsider normalized sexist beliefs toward women. Addressing these areas may improve participants’ engagement with the intervention and may contribute to reducing their heightened risk of IPV recidivism (Travers et al., 2021; Zhu et al., 2024).

Our work is not without its limitations. We identified participants as having a trauma history when they reported having been victims of, or having witnessed, family violence in childhood, as assessed by facilitators during the initial individual motivational interviews. However, IPV perpetrators often struggle to reflect on the impact of ACEs on their present relationship dynamics (Gilbar et al., 2020). Future studies should include comprehensive and validated measures of trauma history, assessed not only at intake but also halfway through and at the end of the intervention, when the therapeutic alliance may be more strongly established (Marshall et al., 2021). Future studies could also complement risk assessment tools with official records of IPV recidivism collected over follow-up periods after intervention completion. Additionally, given the cross-sectional nature of the present study, the associations observed should be interpreted cautiously and do not allow conclusions about developmental, causal, or mediational pathways. Although the present hierarchical analyses help identify variables associated with trauma history and risk of IPV recidivism, they cannot determine whether individual, social-relational, or attitudinal variables mediate the association between trauma history and IPV recidivism risk. Future research should use longitudinal, path analytic, and mediational designs to test the mechanisms suggested by theoretical frameworks, including the general aggression model (Anderson & Bushman, 2002), the intergenerational transmission of violence hypothesis (Voith et al., 2020), attachment theory (Bowlby, 1982), and feminist theories (Gilbar et al., 2020; Lee et al., 2013).

In conclusion, the IPV perpetrators with a trauma history as direct victims or witnesses of family violence in childhood or adolescence may learn that intimate relationships can threaten their safety and security. By identifying the specific risk factors for IPV that are more prominent among the perpetrators with a history of trauma, our findings contribute to a deeper understanding of the question *Wounded boys*,* violent men?* by illustrating how childhood trauma exposure may be associated with trauma-related risk factors at multiple levels in adulthood, which, in turn, may increase vulnerability to IPV perpetration. Therefore, incorporating trauma-informed, motivational-based intervention strategies tailored to their specific needs may enhance the effectiveness of intervention programs for IPV perpetrators and support the development of healthier and safer intimate relationship dynamics (Karakurt et al., 2019).

## Data Availability

The data and research materials are not publicly available due to ethical reasons. Participants are subject to court orders, and confidentiality must always be maintained.
